# Progress in programmatic management of drug-resistant TB, WHO Eastern Mediterranean Region, 2018-2023

**DOI:** 10.5588/ijtldopen.24.0348

**Published:** 2024-09-01

**Authors:** K. Bennani, M. van den Boom, M.G. ElMedrek, Y. Hutin

**Affiliations:** WHO Regional Office for the Eastern Mediterranean Region, Cairo, Egypt.

**Keywords:** DR-TB, MDR-TB, programmatic management, WHO EMR

## Abstract

**BACKGROUND:**

Since 2012, WHO has supported countries in scaling up programmatic management of drug-resistant tuberculosis (PMDT). We assessed progress and challenges to formulate recommendations for improvement.

**METHODS:**

We reviewed the regional Green Light Committee (rGLC)mission reports and analysed data to describe the progression of programme indicators.

**RESULTS:**

The proportion of TB patients initially tested using Xpert MTB/RIF rose from 5% in 2017 to 54% in 2022. Testing for rifampicin-resistant TB (RR-TB) increased from 4% in 2015 to 68% in 2022 among new patients and from 17% in 2015 to 94% in 2022 among those previously treated. Consequently, in 2021–2022, the number of multidrug-resistant (MDR)/RR-TB patients diagnosed increased by 29% and 84% of them were treated, accounting for 22% of estimated cases. By 2023, fourteen countries had implemented all-oral regimens, with three initiating the 6-month bedaquiline, pretomanid, linezolid, and moxifloxacin regimen (BPaL(M)). MDR/RR-TB treatment success increased from 64% in 2017 to 73% in 2020.

**CONCLUSIONS:**

Eastern Mediterranean Region countries progressed in PMDT using Xpert MTB/RIF, increased diagnosis and treatment of MDR/RR-TB patients using all-oral regimens, and improved treatment success. They must now enhance diagnostic capacity using WHO-recommended diagnostics, decentralise services while integrating them into primary health care, and prioritise the BPaL(M) regimen.

## RÉSUMÉ

**CONTEXTE:**

Depuis 2012, l'OMS aide les pays à intensifier la prise en charge programmatique de la TB pharmacorésistante. Nous avons évalué les progrès et les défis afin de formuler des recommandations d'amélioration.

**MÉTHODES:**

Nous avons examiné les rapports de mission régionaux du Comité Feu Vert (rGLC) et analysé les données pour décrire la progression des indicateurs du programme.

**RÉSULTATS:**

La proportion de patients atteints de TB initialement testés à l'aide d'Xpert MTB/RIF est passée de 5% en 2017 à 54% en 2022. Le dépistage de la TB résistante à la rifampicine (TB‐RR) est passé de 4% en 2015 à 68% en 2022 chez les nouveaux patients et de 17% en 2015 à 94% en 2022 chez ceux précédemment traités. Par conséquent, en 2021–2022, le nombre de patients multirésistants (MDR‐TB, pour l’anglais, « multidrug‐resistant TB ») /RR‐TB diagnostiqués a augmenté de 29% et 84% d'entre eux ont été traités, ce qui représente 22% des cas estimés. En 2023, 14 pays avaient mis en place des schémas thérapeutiques entièrement oraux, dont trois ont instauré le régime de 6 mois à base de bédaquiline, de prétomanide, de linézolid et de moxifloxacine (BPaL(M)). Le taux de réussite du traitement de la MDR/RR‐TB est passé de 64% en 2017 à 73% en 2020.

**CONCLUSIONS:**

Les pays de la region de la Méditerranée orientale ont progressé dans la PMDT à l'aide d'Xpert MTB/RIF, ont augmenté le diagnostic et le traitement des patients atteints de MDR/RR‐TB à l'aide de schémas entièrement oraux et ont amélioré le succès du traitement. Ils doivent maintenant renforcer leurs capacités de diagnostic en utilisant les diagnostics recommandés par l'OMS, décentraliser les services tout en les intégrant dans les soins de santé primaires, et donner la priorité au régime BPaL(M).

In 2000, the WHO and its partners established the Green Light Committee initiative (GLC) to address the lack of access to quality-assured second-line anti-TB drugs for MDR-TB patients, which was an obstacle to MDR-TB management worldwide.^1^ By the end of 2009, globally, 63,000 patients received treatment for MDR-TB in 71 countries, and the national TB programmes integrated the clinical and programmatic management of MDR-TB as a part of their national strategies. In the same year, the Global Fund to Fight AIDS, Tuberculosis, and Malaria (The Global Fund) started to fund the GLC initiative to achieve universal treatment for MDR-TB.^1^ Globally, in 2019, the Global Burden of Disease (GBD) project estimated that 1.27 million deaths (95% uncertainty interval [UI] 0.911–1.71) were attributable to antimicrobial resistance (AMR). In 2019, GBD also estimated that TB was responsible for 100,000 deaths associated with AMR.^2^ In 2022, WHO estimated that 410,000 (95% [UI] 370,000–450,000) MDR/RR-TB incident cases occurred, leading to 160,000 deaths (95% UI 98,000–220,000.^3^ The proportion of MDR/RR-TB was 3.3% (95% UI 2.6–4.0%) among new TB cases and 17% (95% UI 11–23%) among those previously treated. Since 2019, WHO has released updated consolidated guidelines on drug-resistant (DR-TB) treatment, featuring improvements in treatment options. The 2022 guidelines included new recommendations for a 6-month all-oral BPaL(M) regimen.^4^ These improve treatment outcomes and quality of life.^5–7^ In 2023, the political declaration of the High-Level Meeting on the Fight against TB pledged to accelerate progress towards reaching approximately 45 million people with TB with life-saving treatment,^8^ including 1.5 million people with DR-TB who need additional efforts to ensure access and expansion of WHO-recommended diagnostics and shorter regimens.

In 2011, WHO and its partners decided to decentralise GLC activities to the six WHO regions and set up regional committees (rGLCs^9^) in a phased manner to scale up the programmatic management of drug-resistant TB (PMDT) and provide closer support to WHO member states. In 2012, this decentralisation was implemented in the WHO Eastern Mediterranean Region (EMR). In 2022, in the EMR, the WHO estimated that 24,000 MDR/RR-TB incident cases occurred (95% UI 16,000–32,000),^10^ accounting for 6% of the global estimate; 87% of the estimated cases were in three countries: Pakistan (63%), Afghanistan (15%) and Somalia (9%). In the same year, the WHO estimated that 2.7% (95% UI 0.76–4.6) of new TB patients and 8.4% (95% UI 1.7–15) of those previously treated were MDR/RR-TB.^11^ In 2022, 11 of the 22 EMR countries receiving rGLC support were dependent on external financing – mainly The Global Fund – for PMDT, including procurement of second-line drugs and laboratory commodities. These countries accounted for 98% of the estimated MDR/RR-TB (Afghanistan, Djibouti, Iraq, Jordan, Lebanon, Morocco, Pakistan, Somalia, Sudan, Syria, and Yemen).

From 2012 to 2023, WHO EMR provided technical support to countries through the rGLC mechanism, improving the quality of services offered to DR-TB people. This increased access to WHO-recommended diagnostics and roll-out of all-oral treatment regimens facilitated the decentralisation of PMDT services, thus making them more people-centred. Overall, case detection and treatment outcomes improved. We assessed the progress and challenges in PMDT in the EMR to document achievements, share lessons learnt and formulate recommendations for programme improvement.

## METHODS

### Documents review

We collected data on the estimated number of MDR/RR-TB cases, the number of TB patients initially tested with WHO-recommended diagnostics and those tested for RR, the number of MDR/RR-TB diagnosed and treated, the number of MDR/RR-TB successfully treated, those who failed the treatment, those who were lost-to-follow-up and those who died. The data source included the 2023 WHO Global TB Report^11^ and TB country profiles. We extracted information on the diagnostics algorithm and treatment regimens adopted from the national TB and DR-TB management guidelines in EMR countries. This retrospective study did not require ethical approval using routine data reported in the Global TB Report and country profiles.

### rGLC missions

We conducted rGLC missions annually in The Global Fund-supported countries to assess PMDT progress and formulate recommendations to strengthen responses. The terms of reference of these missions were aligned with the milestones of the national strategic plans, The Global Fund grant implementation, and WHO guidelines. Technical support addressed case-finding strategy, diagnosis, treatment and model of care, management in children and high-risk groups, monitoring and evaluation, infection prevention and control, active drug safety management, procurement and supply chain management. Missions also contributed to advocacy by sharing reports and key recommendations with senior officials in the Ministry of Health, partners, and other stakeholders.

### Data analysis

We analysed the data collected to calculate the proportion of diagnosed TB patients initially tested with WHO-recommended diagnostics. We plotted the proportion of new and previously treated pulmonary bacteriologically positive cases for RR-TB. We calculated the coverage of MDR/RR-TB testing, detection and treatment and expressed them as percentages. For 2015 to 2022, we plotted the treatment outcomes of MDR/RR-TB cases. For 2022, we described the cascade of care with estimates of people with MDR/RR TB, those diagnosed, those enrolled on treatment, and those successfully treated. For 2023, we plotted the number of countries meeting selected indicators on access to diagnostics and recommended treatment regimens.

## RESULTS

### TB and RR-TB testing using WHO-recommended diagnostics (Xpert MTB/RIF)

**Figure 1.**
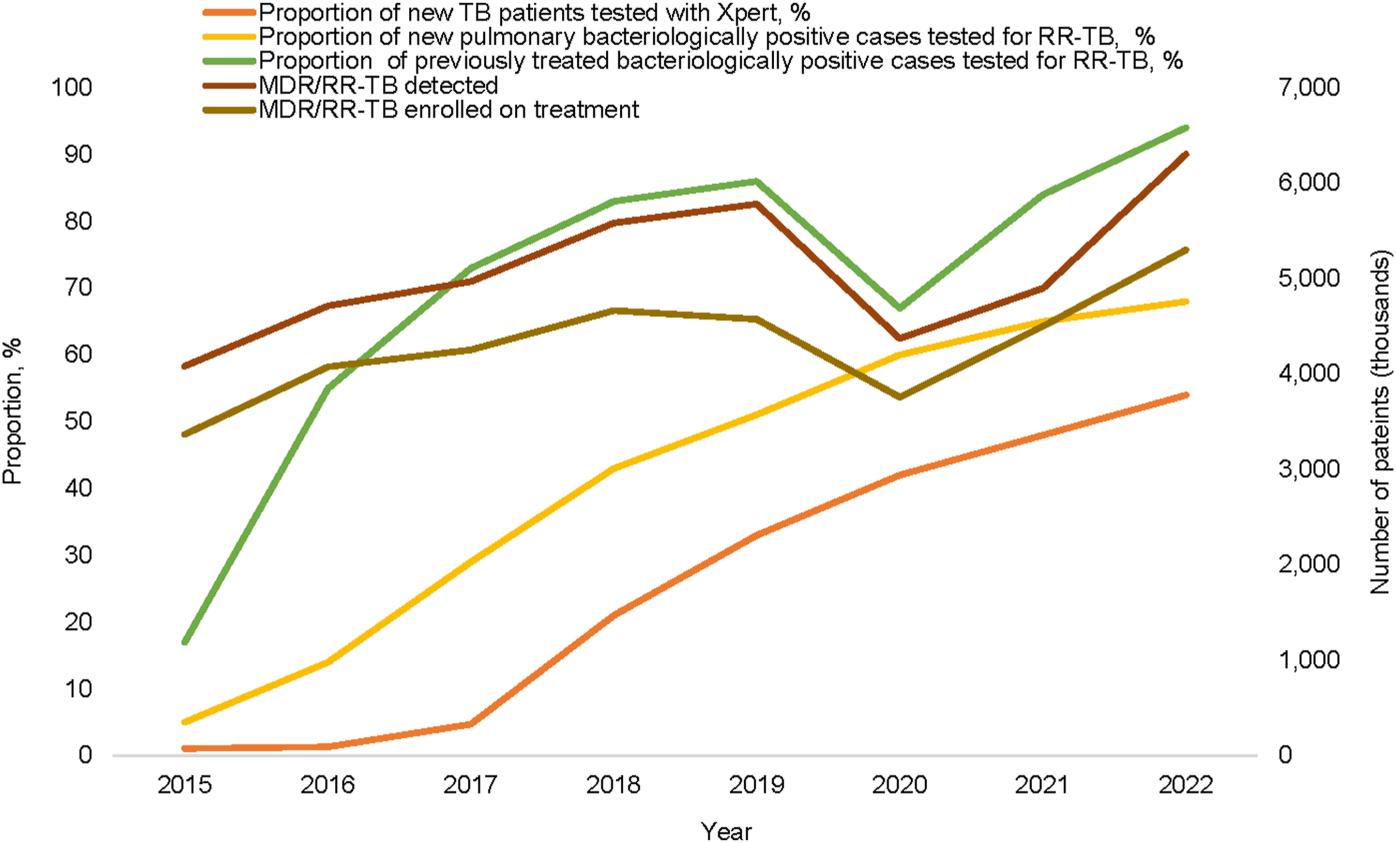
Proportion of diagnosed TB patients tested with Xpert MTB/RIF, those tested for RR-TB and the number of RR/MDR-TB patients diagnosed and treated in EMR, 2015–2022. RR-TB = rifampicin-resistant TB; EMR = Eastern Mediterranean Region.

Between 2017 and 2022, the proportion of TB patients initially tested using Xpert^®^ MTB/RIF (Cepheid, Sunnyvale, CA, USA) increased from 5% to 54%, respectively (Figure 1). In 2022, this proportion varied from 15% in Syria to 63% in Pakistan. Based on bacteriological confirmation, the proportion of people diagnosed with TB increased from 53% in 2017 to 57% in 2021, before a decrease to 56% in 2022.

### RR-TB testing, MDR/RR-TB diagnosis and treatment

Between 2015 and 2022, the proportion of testing for RR-TB increased from 4% to 68% among new pulmonary TB bacteriologically positive patients and from 17% to 94% among those previously treated (Figure 1). As a result, the number of MDR/RR-TB patients diagnosed increased by an average of 9% every year between 2015 and 2019. During the COVID-19 pandemic, it decreased by 25% in 2020 before re-increasing by 12% in 2021 and 29% in 2022 (Figure 1). In 2022, 84% of MDR/RR-TB patients diagnosed were enrolled on treatment, a proportion stable since 2016. The remaining patients were considered as pretreatment lost-to-follow-up. In the region, the number of people treated for MDR/RR-TB increased yearly by 8% on average between 2015 and 2019, decreased by 18% in 2020 during COVID-19 and re-increased by 20% in 2021 and 18% in 2022. From 2018 to 2022, in EMR, 22,800 persons were reported as enrolled on treatment for MDR/RR-TB, representing 18% of the estimated MDR/RR-TB (*n* = 129,000). In 2022, this proportion reached 22% in EMR and 25% in Pakistan, the country with the highest MDR/RR-TB burden. 81% of those treated were in three countries (58% in Pakistan and 11% in Afghanistan and Somalia).

### Access to DR-TB services and WHO-recommended MDR/RR-TB diagnostics and treatments

**Figure 2.**
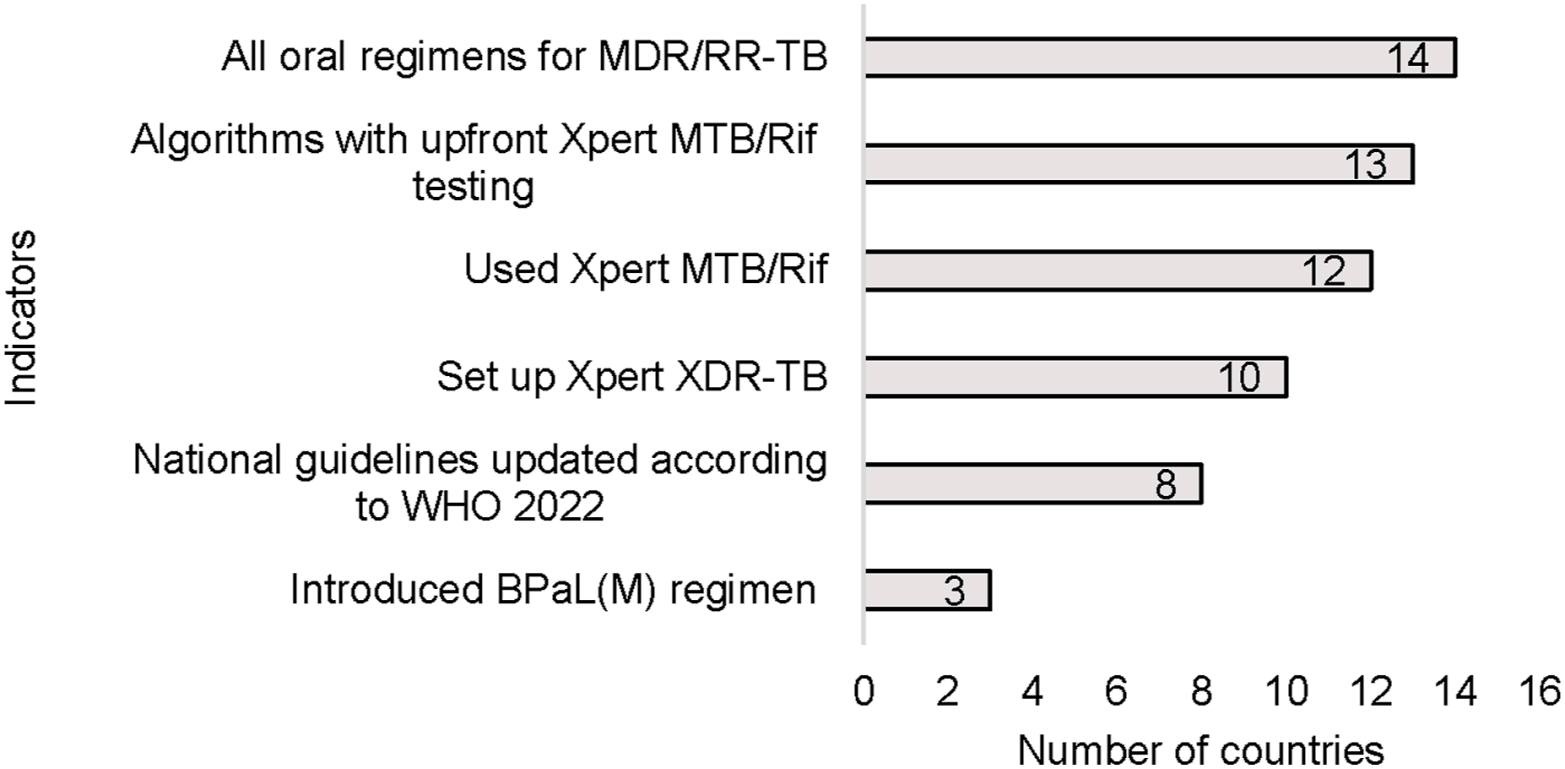
Number and percentage of countries with selected indicators of access to diagnostics and recommended anti-TB treatment regimens, EMR countries, 2023. EMR = Eastern Mediterranean Region.

In 2023, 14 of 22 EMR countries introduced all-oral regimens for the treatment of MDR/RR-TB, and three of them (Pakistan, Iraq, and Yemen) started using the new 6-month, shorter bedaquiline, pretomanid, linezolid, and moxifloxacin (BPaL(M)) regimen (Figure 2). Other countries, including Afghanistan, Jordan, Lebanon, Morocco, Somalia, and Syria, ordered new medicines to start the new treatment regimen in 2024. Thirteen EMR countries updated their algorithms for upfront testing of people with presumptive TB using Xpert MTB/RIF. Eight revised their national DR-TB guidelines according to the 2022 WHO guidelines. Twelve countries set up all the planned Xpert MTB/RIF platforms, thereby improving their diagnostic capacity. Ten countries set up Xpert^®^ MTB/XDR testing (Cepheid) to provide timely resistance tests of fluoroquinolone and other medicines. Fourteen countries adopted the ambulatory model of care for DR-TB management while six Gulf countries centralised service provision.

### Treatment outcomes MDR/RR-TB

**Figure 3.**
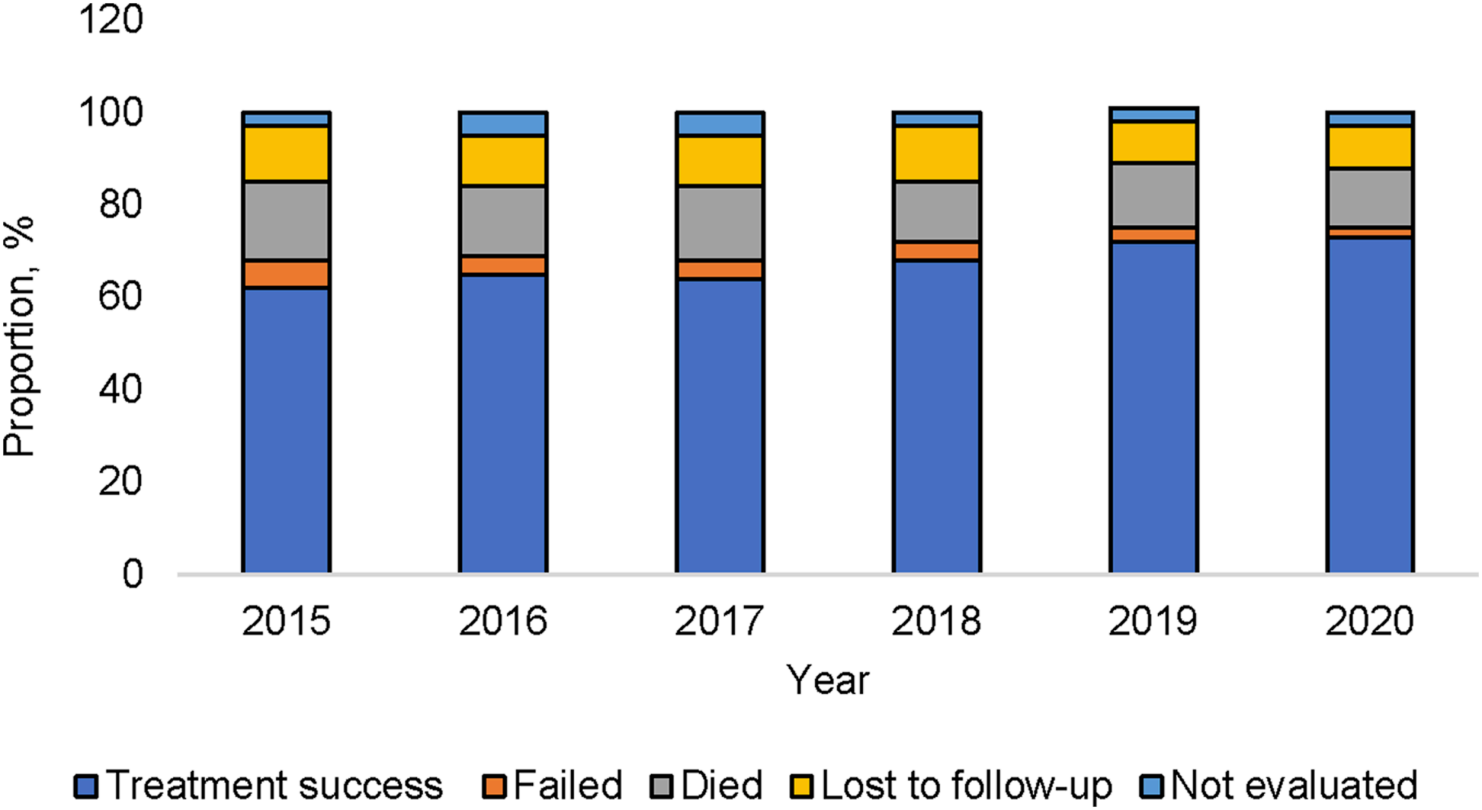
Treatment outcomes of multidrug-resistant/rifampicin-resistant TB cases, EMR 2015–2020. EMR = Eastern Mediterranean Region.

The treatment success for MDR/RR-TB people increased from 64% in 2017 to 73% in 2020 (Figure 3). TB deaths and treatment loss to follow-up decreased since but remained at 13% and 9%, respectively, in 2020.

### Cascade of care of MDR/RR-TB

**Figure 4.**
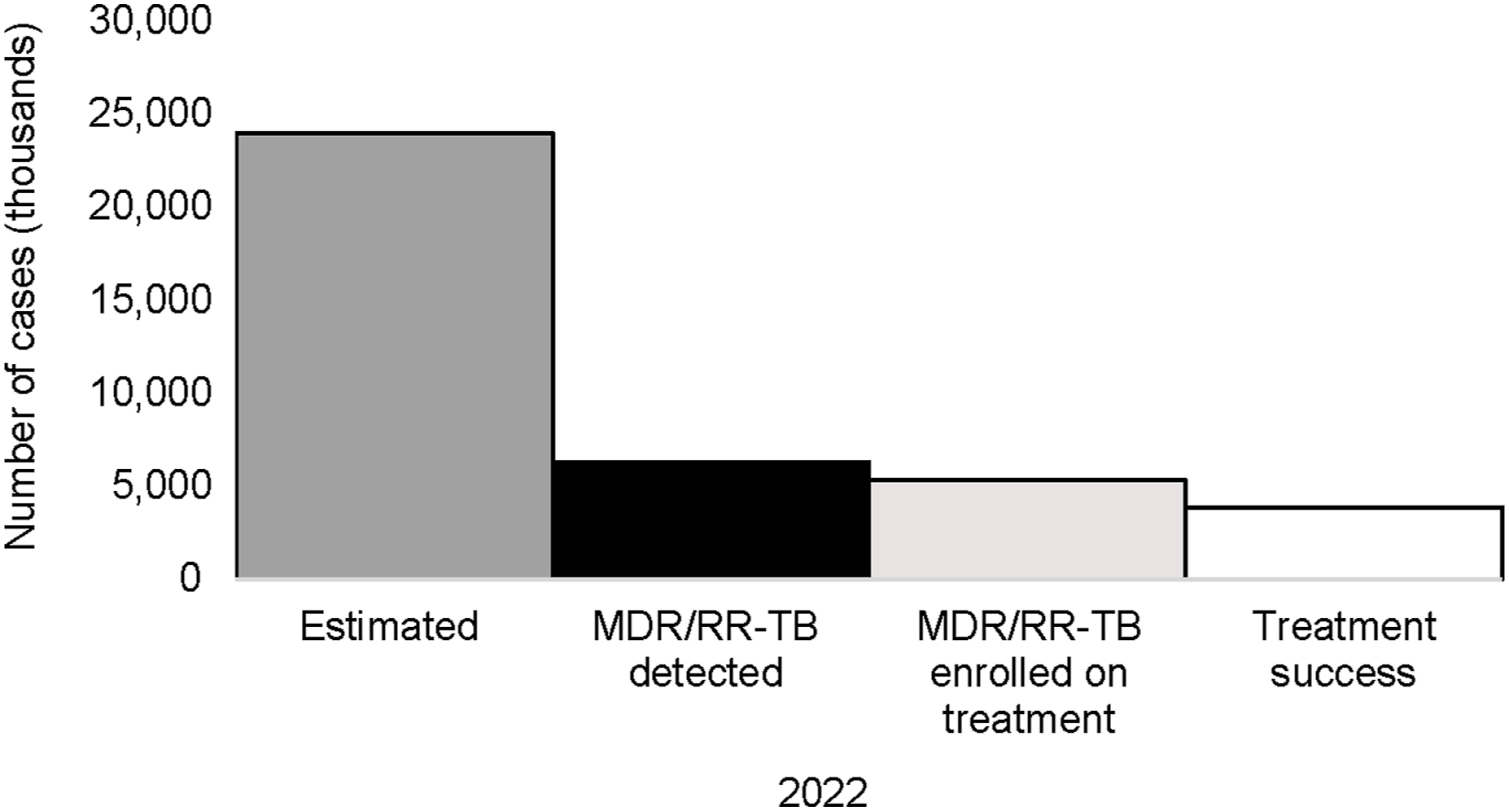
Care cascade for MDR/RR-TB in EMR, 2022 (treatment success of 2020 cohort). MDR-TB = multidrug-resistant TB; RR-TB = rifampicin-resistant TB; EMR = Eastern Mediterranean Region.

The MDR/RR-TB treatment coverage increased from 11% in 2021 to 22% in 2022 (Figure 4). In 2022, three countries accounted for 90% of the regional gap between the estimated number of persons with MDR/RR-TB and those enrolled on treatment: Pakistan (71%), Afghanistan (18%), and Somalia (11%).

## DISCUSSION

EMR countries made progress in testing TB patients using WHO-recommended diagnostics, mostly of Xpert MTB/RIF.^12^ This was made possible by several actions. First, they introduced this test in their TB laboratory network, gradually replacing smear microscopy. Second, they established country-specific mechanisms for specimen transport. Third, they revised their information system to record and report test results. Fourth, they strengthened quality assurance. Fifth, they regularly updated national guidelines and diagnostic algorithms in line with WHO guidelines. Sixth, the 12 Global Fund-supported countries fully implemented their 2023 plans for Xpert MTB/RIF platforms and Xpert MTB/XDR for TB drug susceptibility testing. Progress in the diagnosis systems led to improvement in diagnosis. WHO supported countries through the rGLC missions to evaluate laboratory networks, develop a plan to scale up WHO-recommended diagnostics, update the diagnostic algorithm and closely monitor the implementation of the annual mission recommendations.

In 2022, the proportion of new patients initially tested using Xpert MTB/RIF exceeded the level reported globally (47%).^11^ However, the proportion of bacteriologically positive pulmonary TB (PTB) in the region remained below the 83% global average in 2022.^11^ This apparent contradiction has several explanations. First, in 2022, half of the PTB cases notified in the region were diagnosed on microscopy and not tested on Xpert MTB/RIF. Second, countries did not consistently test smear-negative cases with Xpert MTB/RIF. Third, in case of a shortage of laboratory supplies, they prioritised Xpert MTB/RIF to detect RR-TB in people previously treated for TB. As a result, patients were tested using smear microscopy instead of the more sensitive and specific Xpert MTB/RIF tests. Regional indicators of resistance testing were better. In 2022, in the EMR, the proportion of people tested for RR-TB among previously treated cases exceeded the global proportion (79%). However, RR-TB testing was lower in new pulmonary TB bacteriologically positive cases (73%).^11^ This discrepancy suggests that EMR countries prioritised RR-TB testing in previously treated cases to ensure early diagnosis and treatment for RR-TB.

From 2018 to 2022, in the EMR, the proportion of MDR/RR-TB detected was 18%, lower than the global average (41%).^11^ During the same period, the proportion of the estimated MDR/RR-TB cases enrolled on treatment was lower (21%) than the global reported (38%).^11^ However, the region is making efforts to catch up. Between 2021 and 2022, in the EMR, the number of people diagnosed with MDR/RR-TB increased by 29% and those enrolled on treatment increased by 18%, which exceeded the 6% global increase in detection and the 9% global increase in treatment.^11^ This catch-up can partly be explained by an increase in Xpert MTB/RIF machines and the training of staff in charge of MDR-TB patients in different countries. WHO EMRO contributed to this success by extending its support to countries through rGLC missions. WHO EMRO facilitated in-country training to expand the use of WHO-recommended diagnostics, increase national capacities for case management and introduce the BPaL(M) treatment regimen. However, despite this progress, in 2022, in EMR, the treatment coverage for MDR/RR-TB (22%) remained lower than the global average (43%).^11^ This disparity was due to the low treatment coverage in three EMR-classified high DR-TB burden countries, namely Afghanistan, Pakistan and Somalia, with treatment coverage of 16%, 25%, and 15%, respectively.^13–16^

In 2022, although EMR countries increased MDR/RR-TB diagnosis and treatment enrolment, pre-treatment loss to follow-up remained at 15%. Excessive service centralisation, poor specimen transport systems in emergency countries and limited patient support mechanisms are the main reasons for pre-treatment loss to follow-up. Fourteen region's low- and middle-income countries adopted the ambulatory care model to manage DR-TB. These countries have implemented country-specific models of decentralised management of DR-TB. While decentralised, the DR-TB services are provided vertically with limited integration of services into existing TB health facilities. The six Gulf countries constituted an exception to this approach, where they maintained a centralised service provision.

A lower number of DR-TB patients and the country's preference for hospitalising patients to facilitate the management of adverse reactions to TB medicines explain this difference. Irrespective of the diversity of approaches in the 14 countries and in the Gulf, in the whole of EMR, the MDR/RR-TB treatment success increased from 72% in 2019 to 73% in 2020, higher than the global average (63%) and higher than other WHO regions (from 71% in Africa to 55% in Europe).^11^ In 2020, the loss of follow-up in EMR was the lowest of all six WHO regions and globally.^11^ This improvement in treatment success translates into lower mortality, where TB deaths from MDR-TB decreased by 23% between 2015 and 2020. However, because of the service disruption during the COVID-19 pandemic and limited, decentralised services, in 2020, the MDR-TB death was only the third lowest from all regions, higher than in the Western Pacific (7%) and the Americas (11%) but lower than Africa, South-East Asia (14%) and Europe (15%).^11^

In 2021, all EMR countries, including GFATM-supported countries, introduced the all-oral treatment regimens for MDR/RR-TB, replacing previously recommended regimens with injectable medicines.^17^ The first option was a 9-month all-oral short regimen. The second option was longer, 18–20 months, for people not eligible for the shorter regimen. In 2022, WHO guidelines recommended the 6-month all-oral BPaL(M) regimen.^4^ Subsequently, as of the end of 2023, only Iraq, Pakistan and Yemen introduced BPaL(M) to prioritise it for all eligible patients. The remaining EMR countries are progressing towards using the BPaL(M) regimen, which will improve treatment success and follow-up of MDR/RR-TB people. Through the rGLC mechanism, WHO supported the 12 GFATM-supported countries in rapidly transitioning to the various all-oral regimens. This involved preparing country transition plans, national capacity building and focused dissemination of recommendations in training courses. This technical support improved the clinical and managerial capacities across the entire care cascade for drug-susceptible and DR-TB.

Progress and achievements in PMDT can also improve TB elimination in EMR.^18^ Notably, 11 EMR countries are on track for elimination and have developed a roadmap towards elimination.^19^ This has facilitated the development of country-specific national strategic plans to eliminate TB, including MDR-TB.^20^ The Croatian experience testified to the feasibility of elimination,^21,22^ which reached the pre-elimination threshold for MDR-TB.

Our assessment suffers from two main limitations. First, we could not describe the provision of DR-TB services in the private sector as we could only count DR-TB patients referred to the national programmes for diagnosis and treatment. National programmes may have missed cases diagnosed in the private sector and lost before they started on treatment. This situation may contribute to the low treatment coverage. Second, we had less capacity to assess the implementation of PMDT activities in countries not supported by The Global Fund. Global Fund-supported countries received annual technical assistance missions through the rGLC mechanism. Mission reports led to more updated information. However, we used diversified data sources to maximise the accuracy of the study results.

This review led to four conclusions. First, EMR countries made progress in testing TB and RR-TB using WHO-recommended diagnostics. Second, MDR/RR-TB diagnosis improved, although we continue to miss almost four among five cases. Third, treatment success increased, but centralised services continue to explain pre-treatment loss to follow-up and mortality. Fourth, the region adopted all-oral treatment regimens, but the use of shorter regimens is limited. Based on these conclusions, we proposed several recommendations. First, we should expand the diagnostic capacity using WHO-recommended diagnosis while optimising the existing plans for specimen transport. Second, additional efforts are needed to improve testing and diagnosis of DR-TB and access to treatment. Third, we should decentralise DR-TB services to basic management units while integrating them into primary health care. Fourth, we should prioritise the BPaL(M) regimen with proper management of adverse reactions to TB drugs. Overall, and beyond technical issues, international donors should maintain their financial support to countries in complex emergencies, while other countries should increase domestic funding to sustain the management of DR-TB beyond donors’ support.
